# Melanin in *Fonsecaea pedrosoi*: a trap for oxidative radicals

**DOI:** 10.1186/1471-2180-10-80

**Published:** 2010-03-16

**Authors:** Marcel ML Cunha, Anderson J Franzen, Sergio H Seabra, Marcelo H Herbst, Ney V Vugman, Luana P Borba, Wanderley de Souza, Sonia Rozental

**Affiliations:** 1 Instituto de Biofísica Carlos Chagas Filho (IBCCF), Centro de Ciências da Saúde (CCS), Universidade Federal do Rio de Janeiro (UFRJ), Rio de Janeiro, RJ, Brazil; 2 Laboratório de Tecnologia em Bioquímica e Microscopia, Centro Universitário da Zona Oeste (UEZO), Rio de Janeiro, Brazil; 3 Instituto de Ciências Exatas, Departamento de Química, Universidade Federal Rural do Rio de Janeiro, RJ, Brazil; 4 Instituto de Física, Universidade Federal do Rio de Janeiro, RJ, Brazil

## Abstract

**Background:**

The pathogenic fungus *Fonsecaea pedrosoi *constitutively produces the pigment melanin, an important virulence factor in fungi. Melanin is incorporated in the cell wall structure and provides chemical and physical protection for the fungus.

We evaluated the production of nitric oxide (NO) in macrophages, the oxidative burst and the inducible nitric oxide synthase (i-NOS) activity in interactions between activated murine macrophages and *F. pedrosoi*. Experiments were carried out with or without tricyclazole (TC) treatment, a selective inhibitor of the dihydroxynaphthalene (DHN)-melanin biosynthesis pathway in *F. pedrosoi*. The paramagnetisms of melanin and the TC-melanin were analysed by electron spin resonance. The fungal growth responses to H_2_O_2 _and to S-nitroso-N-acetylpenicillamine (SNAP), a nitric oxide donor, were also evaluated.

**Results:**

Melanised *F. pedrosoi *cells were more resistant to both H_2_O_2 _and NO. Nitrite was not detected in the supernatant of macrophages incubated with melanised fungal cells. However, i-NOS expression was unaffected by the presence of either untreated control *F. pedrosoi *or TC-treated *F. pedrosoi*. In addition, the inhibition of the DHN-melanin pathway by TC improved the oxidative burst capability of the macrophages.

**Conclusion:**

The NO-trapping ability of *F. pedrosoi *melanin is an important mechanism to escape the oxidative burst of macrophages.

## Background

*Fonsecaea pedrosoi *is a soil-borne dimorphic fungus and the major etiological agent of chromoblastomycosis, a chronic disease that can affect immunocompetent hosts. *F. pedrosoi *is usually limited to skin tissue, most commonly on the lower limbs. Infection usually occurs after exposure to the fungus via contaminated soil, splinters or sharp farm equipment, and results in long-term inflammation, suppurative granulomatous dermatitis and fibrosis [1,2]. The affected patients are typically low-income workers that engage in agricultural or manual labour in tropical and subtropical countries. Rarely, *F. pedrosoi *can also cause phaeohyphomycosis, in immunosuppressed patients [3].

The management of diseases caused by *F. pedrosoi *continues to be challenging. Treatment depends on an early diagnosis and the use of systemic antifungal agents and local therapies, including the surgical removal of lesions. The suggested drug interventions are expensive, involving high doses of itraconazole and/or terbinafine (200 to 400 mg and 250 to 500 mg, respectively) daily for over one year. Even with treatment, relapses are common [4,5].

*F. pedrosoi *constitutively produces melanin [6], a pigment that is an important virulence factor in several human pathogenic fungi due to its anti-oxidative, thermostable, anti-radioactive, paramagnetic and metal binding properties. Melanins are present in both prokaryotic and eukaryotic organisms. These ubiquitous dark compounds are formed by the oxidative polymerisation of phenolic or indolic compounds. Melanins have been extensively studied and characterised as negatively charged amorphous compounds with quinone groups, hydrophobic and insoluble in organic solvents [7,8]. Efforts to elucidate the structure of melanins are not yet conclusive due to limitations of the biochemical and biophysical analytical methods available. Electron spin resonance (ESR) can characterise pigments, including melanin, and reveals that a typical melanin spectrum falls between 3300 and 3500 gauss [7-9].

Franzen *et al*. [10,11] reported that *F. pedrosoi *constitutively synthesises melanin in organelles named melanosomes through the DHN-pathway. In *F. pedrosoi*, melanin confers structural integrity as a cell wall constituent and immune protection through antigen masking. *F. pedrosoi *melanin also has anti-phagocytic properties, and is overexpressed during infection [5]. Inside melanosomes, melanin plays a role in the intracellular storage and regulation of calcium and iron ions [11]. The anti-phagocytic properties of *F. pedrosoi*'s melanin were described after interaction with murine macrophages with or without activation with lipopolysaccharide (LPS) and interferon-gamma (IFN-γ) [12,13]. In addition, conidia from *F. pedrosoi *cultures treated with 16 μg/ml of tricyclazole (TC), a DHN-melanin pathway inhibitor, showed a higher susceptibility to activated murine macrophages when compared to untreated fungus [12].

Macrophages are found in granulomas of chromoblastomycosis lesions and may participate in the antigen presentation and innate immune response against *F. pedrosoi *[14]. To contain the growth of pathogens, activated macrophages release oxygen and nitrogen reactive intermediates. NO released by the activated macrophages are fungicidal against *Histoplasma capsulatum *[15], *Cryptococcus neoformans *and *Sporothrix schenkii *[16,17]. The anti-oxidative properties of fungal melanins [18,19], their paramagnetism as revealed by ESR, and the melanin-iron (a known magnetic or paramagnetic metal depending on its oxidation state) association in *F. pedrosoi *raised the hypothesis; the trapping of free radicals by fungal melanin during interactions between macrophages and fungi is a mechanism of oxidative buffering.

The aims of the present work were the following: (I) to characterise the melanin of *F. pedrosoi *by ESR; (II) to investigate the NO production of activated macrophages against *F. pedrosoi *conidia; (III) to detect i-NOS activity during macrophage interactions with fungi; (IV) to evaluate fungal growth after treatment with NO and H_2_O_2_; and (V) to compare these approaches in conidia with or without TC treatment.

## Results

### ESR spectrometry and microwave power saturation of melanins

**Figure 1:**
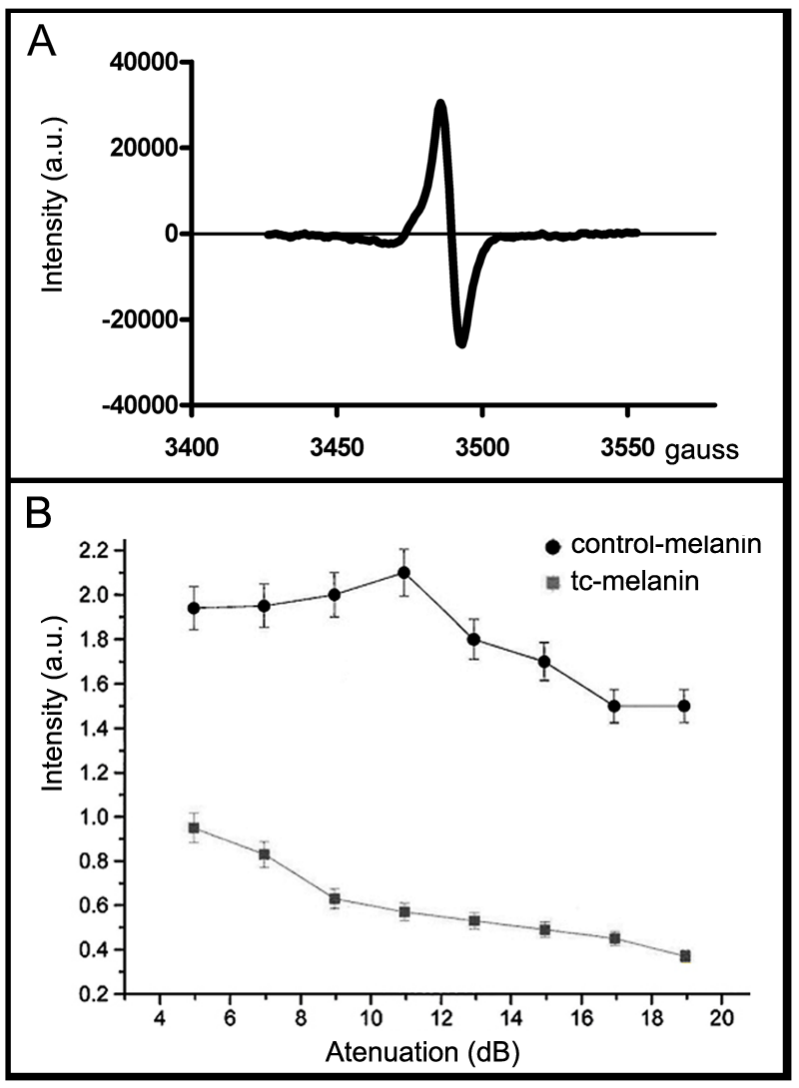
**Electron spin resonance of melanins of *F. pedrosoi***. The ESR spectra (A) of control-melanin or TC-melanin present a single anisotropic line at g = 2.0023. In (B), progressive microwave power saturation experiments show that the paramagnetic centres of these melanins do not saturate under the experimental conditions, and also that the control-melanin sample (black line, circles) has a higher spin relaxation rate than the TC-melanin sample (gray line, squares).

The ESR spectra of the control-melanin and TC-melanin present strikingly similar signals with a peak of 3480 gauss (with respect to line width, line shape, and g value of 2.0023) (Fig. 1A). Progressive microwave power saturation shows that the paramagnetic centres in these melanins do not saturate under the experimental conditions. In addition, these experiments reveal that the control-melanin has a higher spin relaxation rate than the TC-melanin (Fig. 1B). These observations suggest that the control-melanin is a more compact polymer than TC-melanin.

### Oxidative Burst

**Figure 2:**
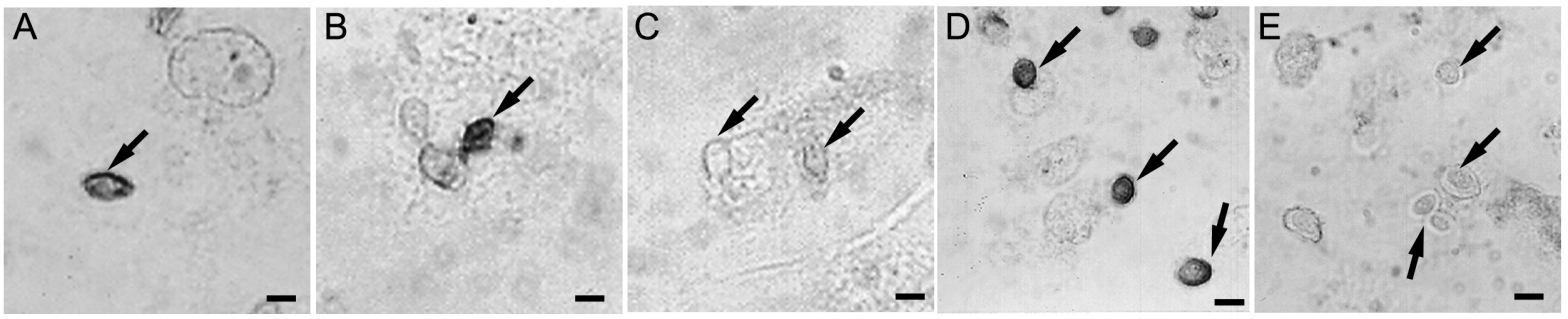
**Light microscopy of the fungal interaction with activated murine macrophages**. Light micrographs of activated murine macrophages after interaction in a 1:10 ratio with: (A) TC-treated *F. pedrosoi *conidia, (B) *F. pedrosoi *conidia grown under low aeration conditions, (C) control conidia of *F. pedrosoi*, (D) *S. cerevisiae *cells and (E) *S. cerevisiae *cells incubated with melanin from *F. pedrosoi*. Fungal cells are marked with arrows. The precipitation of NBT-formazan (dark dye) in response to the oxidative response was observed in A, B and D. Bars = 1 μm

The macrophage oxidative burst was analysed by the NBT assay. The activity of oxidative compounds released by activated macrophages was visualised through the precipitation of NBT-formazan (dark dye) around the fungus in all melanin-deficient systems. This precipitation occurs in response to superoxide molecules near the fungal cell wall (Fig. 2). Formazan precipitation was observed near *S. cerevisiae *(Fig. 2D) and *F. pedrosoi *grown in melanin-deficient conditions, such as with TC treatment (Fig. 2A) or low aeration (Fig. 2B). However, activity of the oxidative compounds was not detected in control *F. pedrosoi *conidia producing regular melanin (Fig. 2C) or *S. cerevisiae *supplemented with *F. pedrosoi*'s control melanin (Fig. 2E).

### i-NOS expression revealed by immunofluorescence

**Figure 3:**
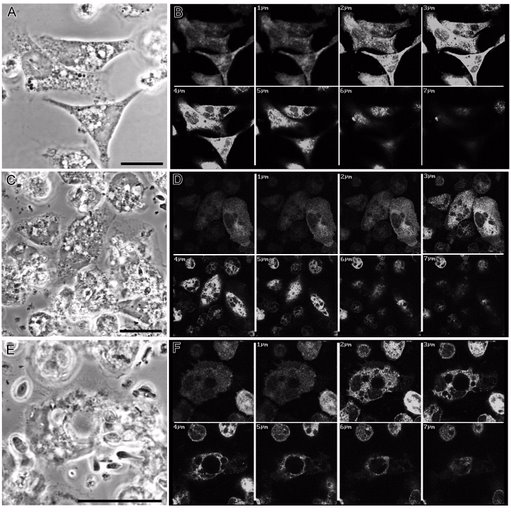
***i*-NOS expression upon fungus-macrophage interaction**. Phase contrast microscopy (A, C and E) and confocal immunocytochemistry (B, D and F) images of activated murine macrophages alone (A-B), activated murine macrophages with untreated *F. pedrosoi *(C-D) or with TC-treated *F. pedrosoi *(E-F). The presence of i-NOS revealed by the anti-i-NOS antibodies conjugated to fluorescent FITC was observed in all experimental conditions tested (B, D and F). Bars = 10 nm.

Immunocytochemistry studies with anti-i-NOS enzymes revealed that these enzymes were active in all models tested: macrophages alone (Fig. 3A, B); macrophages with control *F. pedrosoi *(Fig. 3C, D); or with TC-treated *F. pedrosoi *(Fig. 3E, F). Such data indicate that i-NOS expression was not inhibited in any tested condition.

### Nitrite evaluation

After 24 h of interaction in cultures with *F. pedrosoi *and activated murine macrophages, the nitrite levels were reduced by 91% compared to the amount of nitrite observed in macrophage cultures without fungal interaction (Table 1). A similar reduction was observed when melanin extracted from control *F. pedrosoi *was added to a macrophage culture without fungal cells. Conidia isolated from TC-supplemented cultures yielded a detection of 81% more nitrite compared to non-infected macrophages after 24 h of interaction. Results obtained after 48 hours of interaction suggest a similar concentration endpoint of ~50 μM of nitrite for both macrophages alone or in interaction with TC-treated fungi.

**Table 1:** Nitrite concentration after fungal interaction with activated murine macrophages.

	Nitrite concentration (μM)*	
**Activated murine macrophages**	**After 24 h**	**After 48 h**

**Without fungus**	20.0 ± 0.70	50.0 ± 0.70

**With *F. pedrosoi***	1.9 ± 0.40	4.0 ± 0.28

**With 1 μg/ml of melanin isolated from *F. pedrosoi***	0.9 ± 0.54	1.1 ± 0.14

**With TC-treated *F. pedrosoi***	36.2 ± 1.25	50.0 ± 3.95

*Mean values ± standard deviation recorded after 3 independent experiments.

Molar concentration of nitrite detected after interaction of *F. pedrosoi *or melanin from *F. pedrosoi *with activated murine macrophages for 24 and 48 h.

### Fungal growth after direct activity of oxidative species

**Figure 4:**
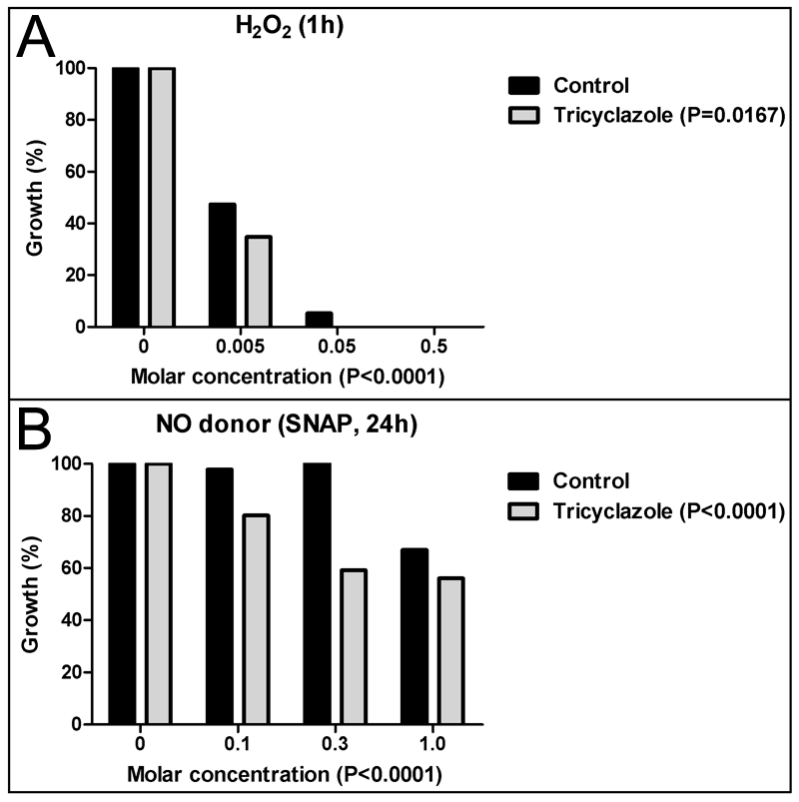
**Fungal growth after exposure to H_2_O_2 _and NO**. Graphic representation of the growth of *F. pedrosoi *with (gray bars) or without (black bars) tricyclazole (TC) treatment after exposure to H_2_O_2 _for 1 h (A), or the NO donor SNAP for 24 h (B). After exposure to H_2_O_2 _or NO, the growth of the TC-treated *F. pedrosoi *was less pronounced than that of the control fungus (P < 0.05). Values are the percentage of growth relative to the control or TC-treated fungi not exposed to H_2_O_2 _or NO.

The growth of TC-treated *F. pedrosoi *significantly decreased in comparison to the control after incubation with either H_2_O_2 _or SNAP (P < 0.05, Fig. 4). Differences were more prominent at concentrations of 0.005 M of hydrogen peroxide and 0.3 M of SNAP.

## Discussion

Fungal melanins are a hot topic among mycologists and have been extensively characterised as virulence factors. Melanin pigments can protect pathogenic fungi from the mammalian host innate immune responses providing resistance: (I) to phagocytosis in *C. neoformans*, *Paracoccidioides brasiliensis*, *S. schenkii *and *F. pedrosoi*; (II) to killing by the host cell in the previously mentioned species as well as in *Aspergillus fumigatus *and *Wangiella (Exophiala) dermatitidis*; and (III) against oxidising agents in *C. neoformans*, *Aspergillus spp*. and *S. schenkii *[8,20].

ESR characterizations of melanins correspond to a peak signal on the spectra near 3355 gauss. These data are coherent among several fungi regardless of the specific melanin biosynthetic pathway or even if the fungus is pathogenic, including *C. neoformans *[21]; *Blastomyces dermatitidis *[22], *P. brasiliensis *[23], *H. capsulatum *[24], *S. schenckii *[25] and *W. dermatitidis *[26], or not, as in the slime mould *Fuligo septic *[27], indicating that, at the molecular level, the structure of paramagnetic center is similar on these melanins.

The ESR characterisation of the samples revealed the presence of paramagnetic centres in both the control-melanin and TC-melanin; however, the control-melanin sample was of a higher intensity indicating that the number of unpaired electrons (free radicals) was higher. Thus, these results indicate that the control-melanin is a polymer with more paramagnetic centres than the TC-melanin.

In the host environment, the expected iron species are diamagnetic Fe(II) and paramagnetic Fe(III). In our experiments Fe(III) was used as a nutrient since we used ferric ammonium citrate as the medium substrate. Fungal melanins are able to reduce Fe(III) to Fe(II), and this oxidative change prevents the formation of oxidative radicals when iron reacts with hydrogen peroxide, thus protecting the fungus from oxidative stress [28]. Cunha et al. [12] demonstrated that untreated *F. pedrosoi *has more abundant and homogeneous binding to cationised ferritin (a Fe(III) complex) on the cell wall surface than fungi treated with TC. At the time, the stronger binding was attributed to more anionic groups on the surface of the control and melanin's affinity to iron.

Experiments with melanin from *C. neoformans *[28] suggests that it acts as a redox buffer, changing its oxidative state according to the chemical stimuli in its environment. Thus, it is possible that melanin maximises its antioxidant potential by reducing Fe(III) to Fe(II), ensuring the balance of its redox chemical microenvironment and minimising the effect of oxidation of fundamental structures on fungal growth.

The novel findings of this work led us to propose that the melanin of *F. pedrosoi *reacts with ferric iron to reduce it to ferrous iron, and maintains this iron-melanin complex as a redox buffer to trap oxidative radicals. This explains the higher growth rate of the control *F. pedrosoi *samples compared to the TC-treated samples following exposure to NO and hydrogen peroxide (Fig. 4), as well as the higher susceptibility of the TC-treated samples to activated macrophages [12].

The progressive microwave power saturation ESR experiments, which varied the power of the microwaves on the magnetised sample, showed approximately a two times higher intensity in the control-melanin samples compared to the TC-melanin samples. According to our hypothesis, this suggests that control-melanin has more self-interaction sites as well as interaction sites for associated structures and therefore is more compact. As indicated by Herbst et al. [29], the profile of progressive microwave power saturation curves of amorphous solids is linked to the effectiveness of spin relaxation pathways for the paramagnetic centre that interacts with its surroundings. Hence, the measure of the progressive microwave power saturation curves for similar paramagnetic centres may provide an indirect indication of molecular arrangements. In this study, the profiles observed for control-melanin (Fig. 1) suggest that it is a more compact polymer than TC-melanin because its spin relaxation rates are faster. Such data are in agreement with the thinner cell wall of untreated *F. pedrosoi *conidia compared to TC-treated *F. pedrosoi *as revealed by freeze-fracture assays [30].

Our data from interaction assays between fungi and activated murine macrophages suggest that melanin is involved in the protection of the fungus against NO. Precipitation of formazan in the NBT assay (Fig. 2) revealed the presence of an oxidative response in the interface between the melanin-free fungi and macrophages. These experiments also showed that the presence of control-melanin (either free in the media or adhered to the fungal cell) decreased NO levels as revealed by its direct correlation to the detected nitrite levels. Further, TC-treatment of *F. pedrosoi *conidia resulted in at least an 80% increase in the amount of nitrite detected after the first 24 h of interaction compared to samples with only macrophages. These data indicate that the inhibition of the melanin pathway, and consequently, the absence of melanin exposed on the cell wall of the fungus, could stimulate the production of NO by activated macrophages.

Fungal glucans, the major component of the fungal cell wall, were previously described to activate macrophages (which express glucan receptors) and promote the synthesis and release of NO [31]. Nimrichter et al. [32] suggested that the removal of melanin from the *F. pedrosoi *cell wall exposes antigens, such as glucans, that were previously masked by melanin. We conclude that the increase of the macrophages' oxidative response after interaction with TC-treated *F. pedrosoi *was probably due to the unmasking of antigens/glucans in the fungal cell wall.

The inhibition of i-NOS expression by pathogens has been reported in other microorganisms, e.g., *Toxoplasma gondii *[33]. Bocca el al. [34] suggested that melanin from *F. pedrosoi *could inhibit NO production in macrophages. However, our experiments suggest that the reduction of nitrite levels after the interaction of macrophages and control conidia was not due the inhibition of i-NOS expression, since its expression was detected in all tested conditions in immunofluorescence experiments. We propose that *F. pedrosoi *melanin acts as a scavenger of oxidative radicals, masking the detection of NO in some systems.

The conversion of L-arginine by i-NOS in the presence of NO requires calcium ions and Fe(III)(in an heme group). Melanin participates in the storage of calcium and iron in *F. pedrosoi*, and therefore it might reduce the availability of such ions in the interaction microenvironment [11,35]. In addition, NO reversibly reacts with both Fe(III) and Fe(II), leaving an electron that could remain trapped in the quinone groups of melanin [8,36].

The assays with the NO donor SNAP and H_2_O_2 _revealed that untreated *F. pedrosoi *grew more than TC-treated *F. pedrosoi*; this suggests a protective function for melanin. In these experiments, our only variables were the *F. pedrosoi *conidia and the oxidative agent. Consequently, in these systems, no other mechanism can occur to inhibit i-NOS production.

## Conclusions

Our data suggest a protective role for *F. pedrosoi *melanin by its direct interaction with NO; the fungal melanin acts as a trap for the unpaired electron of NO, protecting the fungus against oxidative damage. This mechanism impairs the immune system and makes difficult the fungal clearance by macrophages and other phagocytes, which might be related to the recalcitrant nature of chromoblastomycosis lesions and the chronic nature of this disease. Further investigation is necessary to define the structure of fungal melanins and describe putative chemical reactions that could occur in the infection environment, the products of such reactions and possible target sites for the development of new drugs.

## Methods

### Microorganism and reagents

A human isolate of *F. pedrosoi *(5VLP) [37] was inoculated in modified Czapek Dox (CD) liquid media (Sucrose 30 g/L, NaNO_3 _2 g/L, KH_2_PO_4 _1 g/L, MgSO_4_.7H_2_O 0.5 g/L, KCl 0.5 g/L, ammoniacal iron citrate 0.01 g/L), pH 5.5, with shaking at 28°C for five days. TC (kindly provided by Dow AgroSciences, Indianapolis, USA) was dissolved in dimethylsulphoxide (DMSO) and added to cultures at a final concentration of 16 μg/ml to block the DHN-melanin biosynthesis pathway. All other reagents were acquired from Sigma-Aldrich (Brazil), unless otherwise specified. *Saccharomyces cerevisiae *(INCQS 40001, ATCC 2601) was donated by Coleção de Culturas de Fungos of Instituto Oswaldo Cruz, Rio de Janeiro, Brazil.

### Melanin isolation

*F. pedrosoi *melanins were isolated from fungal cultures following incubation with 16 μg/ml of TC (TC-melanin) or without the drug (control-melanin) by an alkali-acid extraction method described elsewhere [6].

### Electron Spin Resonance

After isolation, melanins (10 mg) from *F. pedrosoi *cultures were thoroughly triturated manually in a solid marble mortar with a pestle. The trituration was a necessary step in order to diminish the grain size, which otherwise could lead to preferential orientations and to the observation of artifacts in the ESR spectra. The pigments were analysed by ESR spectroscopy coupled to a spin-trapping analysis. The spectra were acquired at room temperature in quartz tubes on a Bruker ESP 380-E CW/FT spectrometer (Bruker, Germany) operating at X-Band (9.5 GHz). The amplitude modulation was kept constant at 3.0 gauss and low power microwaves were used to avoid saturation. The microwave power saturation experiments were measured between 0.02-200 mW, while all others parameters remained the same. The g factors (the ESR quantity analogous to the chemical shift in nuclear magnetic ressonance spectroscopy), which are related to the magnetic field, were measured upon a diphenylpicrylhydrazyl radical (DPPH) standard, g = 2.0023 [38].

### Conidia Isolation

*F. pedrosoi *cells with or without a treatment of 16 μg/ml of TC were filtered in a 40-60G porous plate filter, followed by conidia recovery by centrifugation (13,600 g, 30 min, 4°C).

### Peritoneal Macrophages

Peritoneal washes with Hanks' Balanced Salt Solution were performed in 2-3-week-old Swiss male mice. Resident macrophages were seeded on glass coverslips in 24-well plates or in Petri dishes for 1 h at 37°C in a 5% CO_2 _atmosphere. Cells were then washed and cultured for 24 h in DMEM containing 10% foetal bovine serum.

### Macrophage Activation and Fungal-Host Cell Interaction

Macrophages were activated with 50 U/ml of recombinant murine IFN-γ and 100 ng/ml of LPS from *Escherichia coli *0111:B4 for 24 h before interaction with the fungus. Interactions were carried out at a 10:1 (fungus:macrophage) ratio for 24 h at 37°C in a 5% CO_2 _atmosphere.

### Oxidative Burst

Conidia were extracted from cultures of *F. pedrosoi *grown in three different conditions: (I) aeration with exposure to light; (II) low aeration in the dark; (III) and supplemented with 16 μg/ml of TC. *S. cerevisiae *was also used in two different conditions: (I) alone as a control or (II) supplemented with 1 μg/ml of melanin isolated from *F. pedrosoi*. The interaction of fungal cells with activated murine macrophages was evaluated on round glass coverslips in 24-well plates using DMEM defined medium supplemented with 0.5 mg/ml of nitroblue tetrazolium (NBT; grade 111), for 15 min at 37°C. After this incubation, non-adherent and non-internalised fungal cells were removed by gentle washes with PBS. The coverslips were again incubated in DMEM for 30 min to reduce background signals, fixed using Bouin's solution, dehydrated in acetone-xylol and mounted in Entellan resin. The oxidative response of the samples was scored as positive after the observation of the precipitation of indigo blue (formazan) around fungal cells in randomly chosen fields under a bright field light microscope.

### Nitrite evaluation

NO detection was evaluated indirectly by measuring the nitrite levels in macrophage cultures supernatants after interaction as described elsewhere [39]. Briefly, macrophages and fungi (at a fungus to macrophage ratio of 10:1) were allowed to interact for 24 or 48 h in DMEM at 37°C, 5% CO_2_. Macrophages culture conditions were the following: (I) macrophages cultured alone; (II) macrophages with TC-treated conidia; (III) macrophages with control *F. pedrosoi*; and (IV) macrophages cultured with 1 μg/ml of melanin extracted from *F. pedrosoi*. Supernatant from each well (100 μl) was mixed with an equal volume of Griess reagent in a 96-well flat-bottomed plate. The absorbance at 540 nm was measured with a Dynatech MR 5000 Microplate Reader. The nitrite concentration was calculated from a standard curve of sodium nitrite diluted in DMEM.

### i-NOS expression detected by immunofluorescence

Macrophages before or after interaction with *F. pedrosoi *conidia with or without TC treatment were fixed for 30 min in 3% formaldehyde in PBS. These samples were incubated for 20 min in 50 mM ammonium chloride in PBS and then washed for 10 min in PBS with bovine serum albumin (PBS-BSA). Cells were then incubated for 40 min with rabbit polyclonal antibody for mouse i-NOS (Santa Cruz Biotechnology, CA, USA) diluted 1:100 in PBS-BSA. Cells were washed twice with PBS-BSA and incubated for 30 min with a FITC-labelled goat anti-rabbit IgG diluted 1:200 in PBS-BSA. The cells were then mounted with N-propyl gallate and observed and photographed in a Zeiss confocal laser scanning microscope.

### Fungal growth after treatment with hydrogen peroxide

H_2_O_2 _(Merck, USA) was added directly to control and TC-treated cultures to final concentrations of 0.005, 0.05 and 0.5 M. Conidia (2 × 10^3 ^cells/ml) were incubated in RPMI-1640, for 1 h at 37°C in the presence of the hydrogen peroxide concentrations mentioned above. From each sample, 50 μl were placed in wells of a 24-well plate with 500 μl of CD with 3% agar. The cultures were incubated at 25°C for 10 days.

Fungal growth was measured by calculating the relative size of the colonies per well for each condition. Images of the bottom of the plates were digitalised and processed using ImageJ software [40] for the following parameters: (I) gamma correction to ensure adequate brightness and contrast of the image; (II) a threshold to define the interface between the fungal growth (black) and the background (white); and for (III) the inversion to define the background as black (grayscale value = 0) and the area of fungal growth as white (grayscale value = 255). A constant area with the diameter of a well from a 24-well plate was the template for the measurements of the "Mean Gray Value" on the Image J software. Measurements were the sum of the gray values of all pixels in the selection divided by the number of pixels, revealing the area of fungal growth. In this work the values were expressed as the normalised percentage relative to its control (100% of growth).

### Fungal growth after incubation with a nitric oxide donor

SNAP, a nitric oxide donor, was dissolved in DMSO and added to untreated and TC-treated cultures of conidia (2 × 10^3 ^cells/ml) in RPMI-1640 at concentrations of 0.1, 0.3 and 1.0 mM. These cultures were incubated for 24 h at 37°C. From each condition, 50 μl were plated in one well of a 24-well plate with 500 μl of CD (solid, with 3% agar). Samples were incubated at 25°C for 10 days. The growth area was measured and using the procedure described above.

### Statistical analysis

Graphic and statistical analyses were performed with GraphPad Prism 5.0 (GraphPad Software, USA). The Student's *t*-test was used for experiments with one variable, and results were considered significant if P < 0.0001. ANOVA tests were used for comparing samples in experiments with more than one variable; the results were considered significant when P < 0.05.

## Authors' contributions

MMLC, AJF, SHS, WS and SR conceived of the study and participated in its design and the writing of this paper. MMLC, AJF and SHS performed the experiments with murine macrophages. MMLC and LPB performed the experiments investigating the activity of oxidative species. MMLC, MHH and NVV performed the ESR experiments. All authors read and approved the final manuscript.
